# Society Meetings

**Published:** 1909-11

**Authors:** 


### SOCIETY MEETINGS.

THE McKean County (Penn.) Medical Society held its annual meeting Tuesday, October 19, 1909, at the town hall in Mount Jewett. Officers were elected as follows: president, Dr. H. L. McCoy, of Smethport; vice-president, Dr. McLean, Eldred; secretary, Dr. R. K. Russell, Bradford; treasurer, Dr. J. C. Walker, Bradford; censor, Dr. W. S. Robison, Bradford.

THE Medical Association of Central New York held its Forty-Second Annual Meeting at Auburn, October 19, 1909, under the presidency of M. P. Conway, of Auburn. The following program was presented:

President's address, M. P. Conway, Auburn; Presentation of genitourinary specimens, J. Henry Dowd, Buffalo; Toxins and the liver, Wm. M. Brown, Rochester; Some cases treated by vaccines, Charles O. Boswell, Rochester; Post operative tetanus (report of a case), M. M. Lucid, Cortland; Some desires of the state department of health, Wm. A. Howe, Phelps; Protective appendicitis, Robert T. Morris, New York; Appendicitis, Albert L. Beahan, Canandaigua; Infantile scurvy involving the hip joint, Nathan Jacobson, Syracuse; Important factors in diagnosis and treatment of surgical tuberculosis, Martin B. Tinker, Ithaca; The treatment of high blood pressure and the control of arteriosclerosis, Henry L. Elsner, Syracuse; Icythyosis, A. A. Young, Newark; Notes on nervous and mental manifestations due to arteriosclerosis, F. H. Stephenson, Syracuse; Gun shot lesion of the spinal column, J. P. Creveling, Auburn; The duty of the physician, E. J. Wynkoop, Syracuse; Taxis or operation—which? William S. Chessman, Auburn; Melanotic sarcoma—case—W. L. Wallace, Syracuse.

THE New York and New England Association of Railway Surgeons will hold its nineteenth annual meeting at the Academy of Medicine, New York city, November 16, 17, 1909. A symposium will be presented on the "Causes of Railway Accidents Individualised." The names of prominent lay officials, attorneys, and surgeons from railways all over the country appear on the program, which looks very attractive. All interested in this line of work are cordially invited to attend. Address, George Chaffee, Corresponding Secretary, 338 47th street, Brooklyn, N. Y.

A UNION meeting of the dental societies of the seventh and eighth districts will be held at Buffalo, October 29 and 30, 1909. The program to be observed is as follows: Henry S. Upson,

M.D., Cleveland, Ohio, Professor of Neurology, Western Reserve Medical School, will give an illustrated lecture dealing with the nervous and mental disorders that are due to obscure dental lesions. Discussion opened by A. W. Hurd, M.D., Superintendent of the Buffalo State Hospital for the Insane, and Frank W. Low, D.D.S., Robert J. Reade, M.A., L.D.S., D.D.S., of Toronto, editor of *Dental Practice,* and J. Wright Beach, D.D.S., of Buffalo, Associate Editor of the same Journal, will present a joint paper relating to the Educational and Legal Requirements in Dentistry (a) in the Province of Ontario; and (b) the State of New York. The discussion will be opened by H. J. Burkhart, D.D.S., of Batavia, and George B. Snow, D.D.S., Dean of the Dental Department, University of Buffalo. Marshall Clinton, M.D., Professor of Surgical Pathology, University of Buffalo, Medical Department, will present a paper dealing with Surgical Diseases of the Mouth, with special reference to tumors. W. W. Belcher, D.D.S., of Rochester, has something good—he is not telling us just what. L. M. Waugh, D.D.S., Professor of Dental Pathology, University of Buffalo, Dental Department, will give us the result of two years' scientific research work on Dental Caries. This, too, will be illustrated, and the discussion will be opened by John O. McCall, A.B., D.D.S., of the New York State Societies' Dental Hygiene Council, and F. H. Sibley, D.D.S., of Rochester. J. H. Beebe, D.D.S., of Rochester, an eminently practical man will give a paper upon Practical Dental Points. W. W. Smith, D.D.S., of Rochester, will open the discussion. R. E. Luther, D.D.S., of Batavia, will give a paper bearing upon the Importance of Oral Hygiene and its Influence upon General Systematic Conditions. Discussion opened by Allen Jones, M.D.

A cordial invitation is extended to the members of the medical profession to attend the sessions to be held at the Iroquois Hotel.

---

THE Southern Surgical and Gynecological Association will hold its next annual meeting at Hot Springs, Va., Tuesday, Wednesday and Thursday, December 14, 15 and 16, 1909, under the presidency of Stuart McGuire, of Richmond. Those desiring to contribute papers should address the Secretary, William D. Haggard, Nashville, Tenn. Lewis C. Bosher, of Richmond, is the Chairman of the Committee of Arrangements.

---

THE Medical Society of the County of Erie held its regular quarterly meeting in the hall of the Young Men's Christian Association, Monday evening, October 18, 1909, at 8:15 o'clock, under the presidency of Charles A. Wall.

After regular routine business, the following papers were presented: Otitis media in infants, by J. F. Fairbairn; Surgical tuberculosis, by W. W. Plummer; Late tendencies in the treatment of surgical tuberculosis, by Prescott LeBreton; Commercialism in medicine, by J. V. Woodruff; Routine fecal examinations, by A. L. Benedict.

---

THE Buffalo Academy of Medicine held meetings during the month of October, 1909, as follows:

*Section of Pathology.*—Tuesday evening, October 5. Program: Addison's disease, F. C. Busch; Tuberculin reactions, N. G. Russell; Vaccines, N. M. McLeod.

*Section of Medicine.*—Tuesday evening, October 12. Program: Finkelstein's views on alimentary intoxications in infancy, Irving M. Snow; On adiposis dolorosa and other abnormal fat deposits. (Lantern slide demonstration), Irving P. Lyon; The relation of hepatic sclerosis to alcoholism, A. L. Benedict.

*Section of Surgery.*—Tuesday evening, October 19. Program: The architecture and clothing of the human foot, B. E. MacKenzie, Toronto, Canada.

*Section of Obstetrics.*—Tuesday evening, October 26. Program: Prolapsus uteri with reference to author's perineal operation, Herman E. Hayd. Discussion opened by L. G. Hanley. Troublesome or dangerous symptoms during pregnancy, Irving W. Potter. Discussion opened by P. W. Van Peyma.

